# Assessing Cultural, Religious, and Spiritual Confidence and Perceived Preparedness in Community Palliative and End-of-Life Care: A Service Evaluation

**DOI:** 10.3390/healthcare14111555

**Published:** 2026-06-02

**Authors:** Zoebia Islam, Francesca Horne

**Affiliations:** 1 LOROS Centre for Excellence, Leicester LE3 9QE, UK; francescahorne@loros.co.uk; 2 Department of Population Health Sciences, University of Leicester, Leicester LE1 7RH, UK

**Keywords:** cultural perceived preparedness, spiritual care, palliative care, end-of-life care, communication, bereavement, staff training

## Abstract

**Background:** Cultural, religious, and spiritual (CRS) needs are central to holistic palliative and end-of-life care (PEoLC), yet the confidence and perceived preparedness of community and voluntary sector staff in addressing them remain underexplored. As PEoLC increasingly occurs in community settings, understanding staff preparedness for culturally and spiritually sensitive care is vital. **Objective:** This service evaluation examined CRS perceived preparedness and confidence among staff across Leicester, Leicestershire, and Rutland (LLR), exploring perceived challenges and available resources. **Methods:** A modified Confidence and Perceived preparedness in the CRS Care Survey was distributed to healthcare, hospice, charity, and community staff (May–August 2025). Likert scale data (*n* = 39) were analysed descriptively; qualitative responses underwent thematic analysis using Braun and Clarke’s framework, which was co-produced with stakeholders. **Results:** Staff placed high importance on CRS needs (cultural M = 4.48, SD = 0.61; religious/spiritual M = 4.66, SD = 0.53) but reported lower confidence in the organisational capacity to meet them (M = 3.15 and M = 3.05). Qualitative survey findings showed that staff recognised CRS needs as central to holistic, individualised care, emphasising proactive assessment and avoiding assumptions. Barriers included fear of causing offence, organisational constraints, and challenges in supporting families, alongside concerns about unmet needs. Participants highlighted reliance on informal resources and a clear need for accessible, lived-experience-based training and practical guidance. A prototype CRS resource toolkit, including lived-experience videos and guidance for supporting Muslim patients, was co-developed and reviewed by healthcare, community, and public contributors. **Conclusions:** Staff commitment to CRS-sensitive PEoLC is strong, but practical tools and training are lacking. A virtual CRS toolkit could enhance confidence, communication, and culturally responsive care across multidisciplinary settings.

## 1. Introduction

The cultural, religious, and spiritual (CRS) framework of individuals is widely recognised as a vital component of holistic care, particularly in palliative and end of life settings. For many, CRS plays a crucial role in helping them make sense of their experiences and provides a source of hope and strength during challenging times [1,2]. The existing literature demonstrates the positive impact of strong spiritual and religious support in palliative care [3,4], and this has been reflected in national care quality guidelines [5]. The current national framework for palliative and end-of-life care (2021–2026) also emphasises the importance of addressing cultural, spiritual, and religious needs in supporting individuals to experience a ‘good death’. When these needs are overlooked, it can lead to significant distress for both patients and their families during an already emotionally difficult time.

Despite CRS needs being essential to improving quality of life (QoL) for patients with PEoLC needs [6], the terms regarding CRS needs are used interchangeably in the literature and in healthcare practice [7]. Broadly, cultural needs in PEoLC means “the specific beliefs, values, traditions, and practices-rooted in ethnicity, religion, or community—that shape a patient’s experiences of illness, pain, death, and bereavement” [8]. Religion and Spirituality are more difficult to define, particularly as the two concepts can overlap. Traditionally, religion has been defined as a “system of beliefs and practices that is related to the transcendent, or as a search for significance in ways related to the sacred” ([8] pg. 17), whereas a spiritual need “does not need to be synonymous with religiosity and that spiritual is relating to the search for existential meaning within any given life experience, regardless of practice of faith or religion” ([9] pg. 49). In this study, we wanted to explore the staff perspective toward CRS needs, so open-ended questions were designed to explore their understanding of CRS needs, which further guided the analysis.

The 10 Year Health Plan for England [10] outlines a national commitment to shifting healthcare delivery from hospitals into community settings, ensuring that patients and families receive personalised care in the most appropriate environment. In line with ambitions for holistic and personalised palliative and end-of-life care, increasing numbers of patients are being supported to choose their preferred place of death. However, national data indicate that hospitals remain the most common place of death (42.8%), followed by home (28.4%), care homes (21%), hospices (5.2%), and other settings (2.6%). At the same time, funding for palliative and end-of-life care is becoming increasingly constrained, prompting policymakers to encourage a shift away from hospital, hospice, and care-home-based services toward community-based provision [11]. This policy direction reflects the dual aims of alleviating pressure on overstretched and underfunded services and improving the accessibility and cost-effectiveness of care. Consequently, patients and families are increasingly relying on community organisations and the voluntary sector for support [12].

This expansion of service providers means that individuals at the end of life may be cared for by a diverse range of professionals across multiple settings. Yet the cultural, spiritual, and religious perceived preparedness and confidence of these professionals remain poorly understood, despite their critical role in delivering truly holistic care. Previous research has begun to explore CRS confidence and perceived preparedness in medical staff members in acute hospital settings [6,13,14,15,16]. Findings from studies have led to the development of toolkits for clinical staff and non-clinical staff [6,17,18,19]. However, a significant gap remains in the literature regarding the CRS perceived preparedness and confidence of non-clinical staff, volunteers, and professionals working in community and voluntary sector settings. The barriers these individuals face in delivering CRS-sensitive care and the resources staff draw on to provide “good” CRS-sensitive care remain underexplored, despite their growing role in palliative and end-of-life support. To address persistent disparities in PEoLC, a service evaluation was conducted across Leicester, Leicestershire, and Rutland (LLR). This evaluation examined the perceived CRS perceived preparedness and confidence of staff working across a diverse range of settings, including primary and secondary care, hospices, community groups, and organisations within the voluntary sector. As palliative care continues to shift toward community-based provision, gaining insight into the preparedness of professionals and volunteers to deliver culturally and spiritually responsive care is increasingly essential for ensuring equitable and holistic support at the end of life.

This evaluation sought to explore the challenges faced by staff across various sectors in LLR in meeting CRS needs and aimed to examine how staff navigate and respond to these challenges by drawing on the resources, frameworks, and support available to them in practice.

## 2. Materials and Methods

### 2.1. Study Design

This service evaluation was based in LLR (UK). Leicester city is a super-diverse city, in which 59.1% of the population describe their ethnicity as ‘not white’ [20]. This service evaluation ran between May 2025 and August 2025. As this was a service evaluation, looking at staff member perspectives only, formal NHS ethical approval was not required.

This manuscript reports on three related but analytically distinct components:

1. A mixed-methods staff survey (quantitative items and qualitative open-text responses).
2. Stakeholder and patient and public involvement (PPI) activities that informed survey development and interpretation.
3. A regional consultation workshop that supported the co-development of a cultural, religious, and spiritual (CRS) resource toolkit.

These components are reported separately to enhance analytical clarity and transparency.

### 2.2. Staff Survey

With permission, a modified version of the Confidence and Perceived preparedness in CRS (Cultural, Religious, and Spiritual) Care Survey, originally developed by Gunawardena et al. [6], was employed in this study (See Supplementary Material Section S1). The first section of the survey included both Likert scale items and open-ended questions to assess participants’ perceived understanding of the religious, cultural, and spiritual needs of patients receiving PEoLC, as well as those of their families. The Likert scale items also measured staff members’ perceived confidence and understanding in addressing CRS needs. For example, one item asked: “How often do you consider the cultural needs of people at the end of life and their families through to bereavement?” (1—Never to 5—Very Often). The Likert scale items were substantially revised from the original version by Gunawardena et al. [6] following feedback from the service evaluation stakeholder group, who found the Likert questions unclear and insufficiently applicable to community-based organisations, due to their focus on ‘healthcare staff’. These adaptations ensured that the survey more accurately reflected the contexts and challenges faced by the diverse range of services included in this evaluation.

The open-ended questions were adapted from Gunawardena et al. [6] to better reflect the context of services provided outside acute hospital settings by changing terms like ‘acute hospital trust’ to ‘organisation’. For instance, one question asked: “Please describe how supported you feel within your organisation to meet the religious, cultural, and spiritual needs of patients and their families at the end of life and through bereavement.” Additional open-ended items explored staff concerns, perceived barriers, training needs, and the types of training accessed to support the provision of culturally, spiritually, and religiously sensitive care.

The second section of the survey collected demographic information, including age, ethnicity, religion, and gender. It also gathered data on the type of organisation the respondent worked for and their professional role. Completion of this section was entirely optional, allowing participants to respond only to the first part of the survey if they preferred. The survey was developed in Microsoft Forms, with a QR generated in Microsoft Forms shared via poster.

The finalised survey was piloted with five individuals from five different organisations across the NHS, voluntary, and charity sectors. The pilot participants represented a range of ethnic backgrounds, including White British, Black Caribbean, Black African, and Indian Asian.

Given the extent of adaptation and the absence of formal psychometric evaluation, Likert scale findings are reported as descriptive, item-level data rather than as validated measures of “competence” or “confidence.” Mean scores are presented to summarise response patterns across items, but findings should be interpreted as indicative of participants’ self-reported perceptions rather than as evidence derived from a validated scale.

### 2.3. Stakeholder Group

This project was co-produced with a range of stakeholder representatives from organisations, including voluntary, community, hospice and hospital-based services across LLR. The stakeholder group consisted of 15 individuals representing key partners. As detailed in the previous section, the survey was shaped by contributions from these stakeholder representatives across these organisations. Stakeholder involvement informed the survey design, item adaptation, and interpretation of findings from the CRS survey. The stakeholder input was not treated as primary qualitative data.

### 2.4. Patient and Public Group

The LOROS’ Patient and Public Involvement (PPI) research consultee group also reviewed and commented on earlier versions of the survey, providing feedback on both content and accessibility, particularly in relation to voluntary and community-sector organisations. This group comprised up to 10 individuals from ethnically diverse backgrounds, including members of the public, carers, and bereaved relatives. PPI feedback focused on content relevance, wording, accessibility, and practical implications, rather than generating analytic themes.

### 2.5. Consultations Workshop and Toolkit Co-Development

The consultation workshop functioned as both a co-production and knowledge translation activity. It enabled stakeholders to reflect on emerging survey findings and to directly inform the development of a prototype CRS resource toolkit.

To ensure that the prototype toolkit was genuinely co-produced [21,22,23,24] and responsive to the needs of staff, emerging findings were first shared with service leads across the region through the LLR Palliative Care and End of Life Taskforce. In addition, a wider group of stakeholders—including members of the wider stakeholder group, the Patient and Public Involvement (PPI) research consultee group, and representatives from health, voluntary, and community organisations across the region—were invited to take part in an interactive workshop held in December 2025.

### 2.6. Ethical Considerations

This project was classified as a service evaluation in line with the Health Research Authority (HRA) Defining Research table (October 2022). Its primary purpose was to understand, describe, and improve existing service—specifically, local staff members’ cultural, spiritual, and religious knowledge—rather than to generate new or generalisable knowledge. The project was designed to address the question, “What standard does this service achieve?” and to inform local service improvement only. It did not involve hypothesis testing, the evaluation of a novel intervention, or the generation of findings intended to be transferable beyond the service being evaluated.

Participants were drawn solely from individuals with direct experience of PEoLC services, and the data collected were used to support internal learning and quality improvement across organisations within LLR. In line with HRA guidance, findings are not intended to be applied to wider populations outside this context. As the project was categorised as a service evaluation and was not managed as research, review by an NHS Research Ethics Committee (REC) or HRA Approval was not required. This service evaluation was reviewed by the Research Committee at LOROS Hospice.

All participants were provided with information about the purpose of the service evaluation prior to participation, including details on voluntary involvement, confidentiality, and data use. Completion of the survey was taken as consent, due to only staff members taking part. Data were stored securely on password-protected organisational systems in accordance with local data protection policies.

Verbal informed consent was obtained from all participants during the consultation workshops. Written consent was not required due to the service evaluation nature of the work; however, all participants were informed of the purpose of the activity and took part voluntarily. No direct identifiers were requested; however, open-text responses were reviewed for potential indirect identifiers before analysis and data sharing.

### 2.7. Data Analysis

The survey quantitative data, including demographic information and Likert scale responses, were analysed using SPSS (Version 12) [25]. For the Likert scale items, descriptive statistics such as mean and standard deviation were calculated to assess trends in confidence and understanding. Qualitative data from the open-ended survey questions were analysed using reflexive thematic analysis (RTA), following Braun and Clarke’s [26] six-phase framework. Analysis was conducted using NVivo (Version 14) [27] and was primarily inductive, allowing themes to be generated from participants’ accounts rather than being mapped onto a pre-existing coding framework, while remaining informed by the study’s focus on CRS care.

Two researchers (FH and ZI) completed the analysis, with FH undertaking familiarisation with the data and generating initial codes across all open-ended responses in the survey. Themes were developed reflexively and iteratively, with attention to patterns of shared meaning across responses rather than frequency. Each time a new theme was generated, FH scanned through the open-ended responses to ensure no data were missed. ZI acted as a critical friend, reviewing and developing codes and themes to support reflexive discussion, challenge assumptions, and refine interpretation. Coding agreement was not sought, consistent with reflexive thematic analysis.

An iterative process was used to refine coding, with frequent review and discussion between both researchers. Preliminary themes and subthemes were then shared with service leads and members of the stakeholder and Patient and Public Involvement (PPI) groups during a consultation workshop. Stakeholder and PPI feedback was used to support interpretation, clarify meaning, and refine the practical relevance of themes, rather than to determine analytic validity. This stage informed both the final thematic structure and the development of the CRS resource prototype toolkit.

These insights were incorporated into the final thematic structure, ensuring that the analysis reflected both the dataset and stakeholder perspectives. In line with reflexive thematic analysis, claims of data saturation were not used as a marker of quality. Instead, analysis sought thematic sufficiency, whereby themes were judged to be conceptually robust, coherent, and adequately grounded in the dataset to address the study aims. The final themes were reviewed in relation to the full dataset, the wider literature, and the study’s service-evaluation context. Qualitative data are presented using illustrative quotations, accompanied by participants’ professional role and organisation type where available; however, not all participants disclosed demographic or occupational information, and identifiers are therefore included only where this information was provided.

### 2.8. Participant Sample and Recruitment

Both purposive and convenience sampling techniques were used to recruit participants. The survey was open to individuals from any professional background, provided they were working in, or supporting individuals with, palliative or end-of-life care needs (see Table 1 for inclusion criteria). Recruitment was supported by a stakeholder group comprising 15 members from 15 different organisations across NHS services, the charity sector, local authorities, and voluntary and community-based organisations.

Approximately 45 organisations and services were approached, including NHS Trusts (acute and community), independent and charitable hospices, local authorities, voluntary and community-sector organisations, faith-based and interfaith groups, carer support services, bereavement charities, community advocacy organisations, and groups supporting minoritised communities (including women’s organisations, LGBTQ+ organisations, and ethnically diverse community groups).

Organisations were identified through existing professional networks, local end-of-life care partnerships, voluntary-sector infrastructure bodies, and community engagement contacts. Initial contact was made via email or through named organisational leads, with invitations circulated internally where appropriate. Participant invitations were shared through staff mailing lists, volunteer networks, and informal organisational briefings. Due to this distributed approach, it was not possible to determine the number of individuals who received the invitation or met eligibility criteria, and therefore a response rate could not be calculated.

Inclusion criteria were intentionally broad. Participants were eligible if they were aged 18 years or over and were currently working or volunteering in roles related to end-of-life care, bereavement support, community support, or related services within the study geography. No exclusions were applied based on professional discipline, contractual status, or length of experience, reflecting the aim of capturing a wide range of perspectives, particularly those that are often under-represented in end-of-life care research, including non-clinical and voluntary-sector roles.

## 3. Results

### Participant Characteristics

In total, 39 staff members from voluntary, community, hospice, and healthcare organisations participated in the study. Participants included healthcare professionals (e.g., doctors, nurses, and physiotherapists), as well as staff working within charity organisations, voluntary services, and day hospices. For the purposes of this paper, the collective term staff is used, as not all participants disclosed the specific type of organisation in which they were employed.

Of the 39 participants, 23 completed the demographic section, resulting in item-level missingness for demographic variables. Among those who provided demographic data, participants ranged in age from 29 to 65 years (Mean = 44.6, SD = 14.0) and were predominantly female (*n* = 21). All respondents who completed the demographic section identified as being from a White ethnic background. A range of religious affiliations was reported, with Christianity being the most common (*n* = 10), followed by no religion (*n* = 8). Other responses included Buddhist (*n* = 1), Agnostic (*n* = 1), Spiritualist (*n* = 1), and Pagan (*n* = 1), with one participant preferring not to disclose their religious affiliation.

Participants reported working across a variety of organisational types. Among those who provided this information, the majority were employed within healthcare organisations (*n* = 14), followed by registered charities (*n* = 6), other types of organisations (*n* = 2), and one participant who preferred not to answer. In terms of professional role, the largest group identified as nurses (*n* = 11), followed by medical staff (*n* = 7), other clinical staff (*n* = 4), and spiritual workers (*n* = 1). Demographic and role-related characteristics are summarised in Table 2.

Although participants represented a range of professional roles and organisational contexts, the available demographic data indicate that the disclosed sample was not ethnically diverse. Furthermore, as demographic information was not provided by 16 participants, it is not possible to determine whether the full sample reflected greater ethnic or socio-cultural diversity. Accordingly, references to participant diversity in this paper relate primarily to professional and organisational diversity, rather than demographic representativeness. The findings detailed in the following sections should therefore be interpreted as reflecting the perspectives of staff who participated in the study, rather than as representative of all community, voluntary, hospice, or healthcare staff within Leicester and the surrounding areas.

## 4. Quantitative Findings

Quantitative analysis of the Likert scale responses revealed several key findings regarding staff confidence and perceptions of CRS needs in PEoLC. Table 3 summarises the mean scores and standard deviations for each survey item.

Staff reported a high frequency of considering both cultural (Mean = 4.48, SD = 0.61) and religious/spiritual needs (Mean = 4.66, SD = 0.53) in their practice. The perceived significance of these needs was also rated highly, with cultural needs scoring a mean of 4.82 (SD = 0.38) and religious/spiritual needs scoring 4.84 (SD = 0.36).

Confidence levels varied across different aspects of CRS care. Staff felt moderately confident asking about religious/spiritual needs (Mean = 3.97, SD = 0.74) and cultural needs (Mean = 3.82, SD = 0.88). However, confidence decreased when exploring unfamiliar CRS needs, with scores of 3.71 (SD = 0.91) for religious/spiritual needs and 3.66 (SD = 0.92) for cultural needs.

The confidence of staff in the services provided by organisations were notably lower. Confidence in services to meet religious/spiritual needs was rated at 3.15 (SD = 1.11) and for cultural needs at 3.05 (SD = 1.16), indicating potential gaps in organisational support for CRS care across LLR.

In addition to the Likert scale findings, responses to binary (yes/no) items provide further contextual insight. The majority of participants (33/39) reported being aware of services that support cultural, religious, and spiritual (CRS) needs, most commonly identifying chaplaincy, bereavement services, and palliative care teams. However, fewer participants reported having received formal training in this area (18/39), with the remainder indicating no training or describing learning as informal or experience-based.

Despite this, there was strong interest in further training, with the majority of participants (30/39) indicating that they would attend training sessions if available and several others responding “maybe,” suggesting openness to engagement. Only a small minority reported that training was not required.

Collectively, these findings suggest that while awareness of CRS-related support services is relatively high, formal training provision remains limited, reinforcing the need for more accessible, structured, and practice-relevant training and guidance.

## 5. Qualitative Findings

This section reports themes derived from the survey open-text responses relating to staff understanding of CRS needs in PEoLC. The qualitative results section has been reorganised into three subsections: understanding of CRS needs, barriers and challenges, and resources and training needs. The corresponding themes and subthemes within each section are summarised in Table 4. Feedback from the consultation workshop informed interpretation and prototype toolkit development but is reported separately.

## 6. Qualitative Survey Findings: Understanding of CRS Needs

All 39 participants responded to the first two open-ended questions, which explored their understanding of the cultural and religious/spiritual needs of individuals receiving PEoLC, including the needs of their families during bereavement.

### 6.1. Holistic and Personalised Care: Importance of Cultural and Religious/Spiritual Needs in PEoLC and Beyond

Staff members demonstrated a comprehensive understanding that effective PEoLC through to bereavement must be grounded in recognising each person’s unique identity and needs. This person-centred approach formed the foundation of their understanding across both cultural and religious/spiritual domains, with many staff members accepting that they “try and ensure that they have their needs met in ways that is appropriate to them” (Nurse, Female).

The emphasis on holistic care was particularly evident in how staff described the importance of acknowledging the distinctiveness of each patient and their family. One participant articulated this clearly, stating that meeting both cultural and spiritual/religious needs is essential to delivering appropriate PEoLC: “It’s about recognising the individual as a unique person and understanding what is important to them from a cultural perspective and how that will affect the care they wish to receive.” (Clinical Nurse Specialist, Palliative Care Service). Another echoed this sentiment, highlighting the need for active inquiry: “Everyone is an individual, and questions should be asked to determine whether there are any specific cultural needs at that time for that patient and family.” (Community Nurse, NHS Community Service).

Fifteen staff members specifically described cultural needs as a “broad concept,” encompassing values, decision-making preferences, and expectations around care. This reflects an understanding that cultural identity is not the same for each person but deeply personal and dynamic. Participants recognised that effective care requires engaging with each person’s worldview and understanding how it shapes their experiences and choices at the end of life. Similarly, when exploring understanding of religious/spiritual care, staff members emphasised that such needs are unique to each person and that “It is important to understand what religious/spiritual needs a patient has, also if they would wish for any different practises especially during end-of-life care” (Consultant Nurse, NHS Community Service).

The perspective that staff members view cultural and religious/spiritual needs as an essential part of holistic and personalised care was fundamental across all other themes. This foundational understanding demonstrated that participants did not view cultural and spiritual/religious considerations as additional or optional elements to be addressed only after clinical needs had been met. Instead, they regarded these aspects as integral to the entire care experience, requiring consistent attention and integration throughout the delivery of end-of-life care.

#### Proactive Assessment of Cultural and Religious/Spiritual Needs

A key aspect of providing culturally and religiously or spiritually sensitive PEoLC is actively engaging with patients and their families to understand their specific needs. Staff emphasised the importance of asking rather than assuming, particularly in relation to spiritual and religious needs, recognising that cultural identity does not automatically determine individual beliefs or preferences.

### 6.2. Proactively Asking Patients About Their Cultural Needs

Two staff members identified the importance of proactively asking patients about their cultural needs, as opposed to solely relying on medical notes. In some occasions, one Staff member explained that although a patient’s cultural background might already be documented, it remained essential to “explicitly ask them whether there is something I need to be made aware of in relation to this.” (Consultant, Acute Hospital). This view was also echoed by another staff member who noted that “cultural needs can be wide ranging—asking people what is important to them as they become more unwell can help identify whether faith or culture is part of that”. (Community Nurse, Hospice).

### 6.3. Not Assuming a Person’s Spiritual or Religious Needs

Six staff members explained that it was essential not to assume a person’s spiritual or religious belief based solely on their cultural identity. As one staff member explained “just because someone has a defined religious belief does not mean that their needs are known”. Four participants, two of which were NHS staff members, highlighted the importance of explicitly asking patients and their families about their expectations, including those extending beyond death: “Have they any expectations or wishes they would like to try and achieve as they approach end of life? Can I facilitate in contacting a religious representative during their time with us? What are their wishes after death?”

Participants recognised that religious needs are highly individualised, varying not only between different faiths but also among followers of the same faith. One staff member explained, “Religious needs vary between faiths and even followers of similar faiths may follow or not follow specific tenets. I ask patients if they keep a faith and if so I’ll ask what they need from us to allow them to satisfy their religious needs.” This approach reflects an understanding that religious and spiritual care must be tailored to each person’s interpretation and practice of their faith. As another participant stated, “This is about a person’s interpretation of the faith they follow. It’s important to explore what their faith means to them and how they practice it.”

#### Understanding Cultural Identity of Patients and Families

Twelve staff members identified themes that specifically impacted the cultural needs of patients and their families. These included the recognition that “culture goes beyond religion and ethnicity”, and the “importance of dietary needs seen as vital to culture.” These insights reflect a broader understanding of culture as encompassing everyday practices, values, and expectations that shape how individuals experience care at the end of life.

### 6.4. Culture Goes Beyond ‘Religion’ and ‘Ethnicity’

Participants demonstrated a broad and nuanced understanding of cultural needs in PEoLC, consistently emphasising that these needs extend beyond religion and ethnicity. As one staff member explained, “My understanding of cultural needs is not just about religion, it’s about the values and beliefs which people have,” highlighting the importance of recognising individual preferences and practices.

Staff members articulated a shared awareness that cultural needs encompass more multiple dimensions of identity. As one staff member explained “Cultural needs included needs specific to ethnicity and religion but also sexual orientation and disability…” At end of life. these needs become even more important to a patient and family. This understanding was reflected in the emphasis on creating space for patients and families to observe meaningful practices, such as dietary requirements and social support.

The staff also described the importance of the physical and emotional environment, noting that cultural care can involve “who spends time with that person, who provides care, being able to eat their preferred food, wear their preferred outfits, [and] have particular items around them to create a familiar environment.” These details were seen as vital to maintaining dignity and comfort, especially when patients are away from home.

Importantly, participants stressed that cultural needs are not static or universally defined. One staff member stated, “Cultural needs will vary and it isn’t just based on ethnicity,” while another reflected on the complexity of cultural identity: “Different social cultures may value different approaches to end-of-life care… I had a patient who was ethnically Jewish and wanted to have lots of family around while she was dying… but she didn’t observe any Jewish religious rituals and didn’t self-identify as religious.” This underscores the importance of asking patients directly about their preferences, rather than relying on assumptions based on cultural or religious labels.

### 6.5. Importance of Dietary Needs Seen as ‘Vital’ to Culture

Staff members consistently emphasised the importance of practical actions they could take to support patients and families during PEoLC. A key area identified was the importance of dietary needs, which was seen as central to respecting cultural and personal preferences. Three staff members specifically highlighted the need to ensure patients’ “dietary requirements” were met, recognising this as a meaningful way to uphold dignity and individual values.

*“Representing their dietary needs, language and acknowledging their practices”*

By utilising available resources, staff aimed to honour the wishes of patients and their families, reinforcing a person-centred approach to care that integrates cultural sensitivity into everyday practice.

#### Religious/Spiritual Practices of Patients and Their Families

Fourteen staff members identified that the religious and spiritual needs of patients at the end of life were primarily centred around “rites, rituals, and prayers.”

### 6.6. Rites, Rituals and Prayers

Staff members discussed the importance of religious and spiritual practices in PEoLC, recognising that these needs often become more pronounced as patients approach death. Participants highlighted the diversity of practices across and within faiths, discussing specific examples of religious practices.

*“May have prayers, rituals to complete, items need to have in their possession, cannot assume that they follow any particular path/religious”*

*“Some religions have ceremonies or procedures relating to end of life like Catholic’s having last rites read. Other people may find having visitors from religious chaplains or people from their place of worship comforting. Muslims would opt for positioning towards Mecca at end of life. Muslims need to be buried as soon as possible. Hindu believers would opt for cremation.”*

Staff members emphasised the essential role of religious and spiritual practices in supporting patients’ wellbeing and preserving dignity at the end of life. They described how part of their role involves actively facilitating these needs, recognising that spiritual care is not an optional extra but a core component of holistic palliative care.

*“Enabling a person, and their friends and family, to adhere to religious practices throughout their palliative care journey as well as when they are dying/after a person has died.”*

## 7. Qualitative Survey Findings: Barriers, Concerns and Practical Challenges

### 7.1. Role of Family and Community in Supporting Patients with a PEoLC Need and Their Families Through to Bereavement

An important aspect of delivering holistic and personalised PEoLC was recognising the vital role that friends and family play in supporting the patient, staff members and community staff. Staff identified a range of contributions that families make, particularly in relation to cultural and religious or spiritual care. While families can facilitate meaningful and appropriate care, they may also present challenges that require sensitive navigation. Rather than adopting a deficit-based view that positions families primarily as recipients or in need of support, staff recognised the value of engaging with family members as partners who can provide essential information to enhance care delivery. Several staff members described accessing tools and strategies to support families, which in turn helped improve outcomes for patients.

### 7.2. Role of Friends and Family in Cultural Practices and Decisions

The majority of staff members recognised the essential role that friends and family played in ensuring the patient’s cultural practices are met, from decision making and bereavement supporting to informing staff members of cultural customs. As one staff member noted, “Families (I have known this in Irish families) may opt for an open casket and welcome visitors prior to the funeral” (Bereavement Support Worker, NHS Trust), illustrating how traditions can shape end-of-life experiences and rituals.

Communication preferences also varied, with some families preferring a family-led approach while others prioritised patient-centred communication. Staff recognised that “family involvement in decision-making and bereavement” is central, and support should extend to bereaved families through culturally appropriate counselling or community-led mourning practices. In some cases, families can inadvertently act as a barrier to advance care planning, particularly when they are not yet “ready to discuss prior to death… this can make it harder when they die to ensure their preferences are met” (Community Nurse, Hospice). Hence, facilitating conversations around end of life, in a sensitive way, can improve a patient’s experience of PEoLC. Actively listening to patients and, where appropriate, their families enables the prioritisation of individual and collective values, supporting a collaborative and genuinely person-centred approach to care.

Participants emphasised the importance of initiating early conversations to identify each family’s unique needs, expectations, and preferences regarding end-of-life care. As one participant highlighted: “I think it is important to take these into account as each family has different needs and expectations towards the end of life and death. It is important we know these before to ensure we can involve the correct people and to make sure things are timely if required.” Staff also described facilitating family presence and involvement as desired by the patient, with one noting, “Have family with them and involved as they wish,” and recognising that “cultural traditions and expectations will affect how people are supported by their families and wider support network.”

Practical examples included providing quiet spaces for families, using ‘cuddle beds’ so loved ones can lie together, and even arranging weddings for patients too unwell to leave the hospice. Staff also acknowledged the importance of understanding and respecting beliefs and customs related to death, dying, afterlife, symptom management, communication, rituals, and mourning. As one participant reflected, “It may be that they have an extensive family and they want them to be present. It maybe they do not want any family present. Depending on their background and beliefs or non-belief, it is important to try and understand their needs and wishes and also those of their family members and to respect and support wishes and religious practices.” Staff members particularly highlighted concerns regarding how best to support families. While many acknowledged that friends and family often play a vital role supporting the patient, it was difficult for some staff members to know how to effectively meet the needs of families. This tension was reflected in in the following account:

*“I worry that families do not know what support is available to them or what they can ask for and when I ask a general ‘how can we support you’ or ‘do you or your family member have any religious needs’ families focus is usually on the patient and their physical needs but I am not sure I always take the time I should to make sure families are well supported from a cultural/spiritual standpoint.”*

(Nurse, Hospice)

### 7.3. Role of Friends and Family in Spiritual/Religious Care and Bereavement

Similarly, regarding cultural needs in PEoLC, participants noted that religious and spiritual needs were also significantly shaped by the involvement of friends and family. Several staff members observed that families often relied heavily on faith, particularly during periods of bereavement, and emphasised the importance of ensuring that they were adequately supported and meaningfully “listened to.”

*“The family will relive this time many times and knowing that everything possible is done will help them with their bereavement”*

(Bereavement Support, NHS Trust)

Staff members often spoke about practical ways they could meet religious and spiritual needs.

One commonly cited example was the importance of ensuring the timely release of the body when families requested a prompt burial, recognising this as a significant element of faith-based practice for some communities.

*“We also facilitate the rapid release of a body of some Muslim patients to facilitate the burial of the body within 24 hours when this is requested by the family.”*

(Nurse, Hospice)

### 7.4. Impact of Unmet Cultural and Spiritual/Religious Needs

Participants demonstrated a clear understanding of the significant consequences that arise when cultural and religious or spiritual needs are not appropriately recognised or addressed within PEoLC. Staff recognised that neglecting these aspects can be profoundly distressing for both patients and their families. As one participant reflected, “Unmet religious or spiritual needs can lead to a great deal of distress at an already emotional time for patients and families.” This insight underscores the imperative for proactive, person-centred approaches that engage meaningfully with individual beliefs, values, and practices, rather than assumptions based solely on perceived cultural or religious identity.

Several staff also emphasised the importance of creating space for these rituals and being responsive to individual needs, recognising that religious and spiritual care must be tailored rather than assumed.

*“We try to provide the appropriate spiritual support by contacting different faith leaders when needed. It can also mean saying the rosary with a catholic patient or moving a Muslim patient’s bed so that the head faces Mecca, for example. Many of our patients like to have recorded prayers playing at their bedside or have family members praying with them and we try to facilitate a quiet space for these practices.”*

(Nurse, Hospice)

Despite several staff members acknowledging the essential role they play in supporting and upholding any religious or spiritual practices for the patient and their family, many also highlighted the practical challenges involved in doing so. Although staff tried to proactively meet for example, organising religious leaders or contacting chaplaincy team, such efforts were often characterised as difficult to implement in practice. These challenges were reported as being particularly acute when support was required “out of hours,” when access to faith representatives or specialist services could be limited.

*“It can be difficult to access some religious leaders especially at night and now our chaplaincy service is depleted that is not going to get any easier.”*

### 7.5. Fear of Causing Offence

Owing to the staff members’ recognition and understanding of cultural and religious/spiritual needs, 10 staff members highlighted a key concern was unintentionally offending patients and their families. This stemmed from concerns about misunderstanding religious terminology or not knowing how to initiate conversations about culture, religion, or spirituality.

*“My main concern is about unintentionally causing offence or distress by asking questions in an insensitive way, especially during such a vulnerable time.”*

(Advanced Clinical Practitioner, community hospital)

*“Not fully understanding the religion or wording used and therefore showing ignorance to their faith or culture in an already upsetting time.”*

(Diana Nurse, children’s community service)

*“I think it’s common that healthcare professionals feel worried they might say the wrong thing, that asking questions may seem intrusive or even ignorant or that it is not within their role.”*

(Doctor, hospice)

## 8. Resources, Training Needs and Perceived Support

### 8.1. Use of Existing Resources

To minimise the risk of causing unintentional offence, staff members sought resources already available to them to educate themselves about different religious/spiritual and cultural “dos and don’ts” when providing PEoLC.

These were split into four categories: 1. professional teams and services (i.e., chaplaincy, bereavement services, end-of-life champions, Spiritual Gate); 2. self-directed learning and research (e.g., reading information, speaking to scholars, own reading, self-guided research); 3. training and education resources (e.g., in-house palliative care training, yearly end of life palliative care study, LOROS Hospice training courses) and 4. informal practice and peer support (e.g., asking questions, talking to colleagues).

### 8.2. Need for Accessible Training and Guidance

Across the dataset, twenty staff members emphasised the need for training grounded in lived experience, as well as a toolkit offering a “broad overview of traditions required for specific religious needs” (Nurse, Hospice). This highlights a broader concern that current training is often inconsistent, ad hoc, or lacking in practical relevance. Some staff members expressed uncertainty about what resources are available or appropriate, describing current provision as “very ad hoc” or responding “not sure” when asked what support exists, indicating a lack of clarity and consistency in access to guidance.

Participants were not simply asking for more information but for training that supports meaningful engagement with patients and families. In particular, there was a clear preference for learning directly from those with lived experience or from individuals representing different cultural and religious backgrounds. As one participant noted, it would be valuable to have “face to face or video training from people of different cultures/religious/spiritual backgrounds explaining their needs,” while another emphasised the importance of “formal taught and interactive session[s]… and examples of lived experiences.” This reflects a shift away from static or checklist-style knowledge towards a more relational and interpretive understanding of care, where staff can better understand not only what practices are important, but why they matter to individuals and families.

Accessibility and flexibility in training delivery were also emphasised. Participants highlighted the value of online resources that could provide a “general overview,” while also expressing a need for more in-depth, interactive formats such as study days or workshops: “I think this could be a whole day training… these areas are often overlooked and rushed into a few hours.” At the same time, there was recognition of the realities of busy clinical environments, with suggestions that training should be delivered in “bite size pieces for busy hospital staff.”

There was also strong support for embedding CRS training into routine professional development, with participants calling for “mandatory training,” “regular updates,” and “ongoing support and annual training.” This suggests that staff view CRS related knowledge not as a one-off learning need but as an evolving and essential component of practice requiring continuous reinforcement.

Alongside training, participants highlighted the need for clear and accessible guidance to support decision making in practice. Suggestions included a “well written toolkit,” “checklist[s] to prompt,” and “links to trusted sites,” as well as improved access to trained interpreters who are confident in “medical conversations around death and dying.” These findings indicate that staff require not only knowledge but practical, easily accessible resources that can support them in real-time clinical situations.

Collectively, these responses suggest that improving CRS care requires a shift from fragmented and informal learning towards more structured, accessible, and practice-oriented training and guidance, supported by organisational commitment and integrated within everyday care delivery.

### 8.3. Consultation Workshop and Prototype Toolkit Feedback

Twenty-seven participants attended the workshop, where they were presented with emerging survey findings and a prototype CRS resource. The prototype CRS toolkit focused specifically on supporting Muslim patients and included: (1) guidance on Islamic cultural and religious practices at end of life; (2) lived-experience video content; (3) practical communication guidance; and (4) signposting to local support services.

Participants were then asked to respond to four structured questions focused on the cultural, religious, and spiritual aspects of the proposed prototype toolkit:

1. What practical challenges do you face in meeting cultural, religious, and spiritual needs at the end of life?
2. What resources do you currently use?
3. What resources or training do you feel are needed to address these challenges?
4. What should a toolkit for this purpose contain and look like?

The workshop provided an opportunity for participants to review the developing prototype toolkit, offer feedback, and contribute their insights. The workshop enabled participants to review emerging findings and provide structured feedback on the prototype toolkit.

Participants supported the development of practical resources that provide a broad overview of cultural, religious, and spiritual needs, while emphasising that such resources should not encourage assumptions about individuals.

There was strong support for incorporating lived-experience content, including video-based materials, to enhance relevance and accessibility. Participants also highlighted the value of including clear guidance on accessing local services and community resources.

In addition, participants argued for the creation of a staff forum to facilitate ongoing learning, reflection, and sharing of practice across organisations.

## 9. Discussion

The findings presented in this paper are results from a service evaluation exploring the perspectives of staff members, working across a range of sectors, who support people accessing PEoLC and their families through to bereavement. Overall, survey participants demonstrated a good understanding that cultural and spiritual needs form an essential part of holistic care. This understanding intersected across several themes, shaping staff perceptions of both the challenges and opportunities associated with delivering high-quality CRS support within PEoLC contexts. In alignment with these findings, participants described a range of strategies they employed to recognise and address CRS needs. These included undertaking proactive assessments, engaging with patients’ friends and family, and adapting care practices to reflect individual beliefs and values. Staff consistently noted that unmet CRS needs could lead to considerable distress for both patients and their families, particularly at the end of life and during the bereavement period.

In addition to these themes, several findings emerged uniquely within each domain of CRS needs. Within the cultural domain, the majority of staff members emphasised that culture extends beyond religion and ethnicity, viewing it as deeply personal and unique to each individual. This perspective reflects a broader understanding of cultural identity as encompassing personal values, life experiences, and social contexts. A minority of staff identified the provision of appropriate dietary options as one practical way in which they sought to ensure that CRS needs were met.

In the domain of religious and spiritual needs, most staff members associated these with rituals, rites, and prayers. However, they also acknowledged that these practices are not universally applicable and should be tailored to the individual. This personalised approach highlights the importance of avoiding assumptions and engaging with the person and their family to ensure CRS needs are met. Staff members described several resources and approaches they use to support this, but most importantly, they emphasised the need for a prototype resource toolkit. This would ideally include a broad overview of traditions, access to training that incorporates lived experiences, and guidance on how to access community resources such as interpreters.

### 9.1. Implications for Practice

The findings support previous research showing that staff recognise the importance of assessing and meeting the cultural, religious, and spiritual (CRS) needs of patients and their families [6]. Consistent with the existing literature, staff also reported facing multiple barriers in practice, including language barriers, prejudice, and organisational constraints [6,15,28].

However, unlike some previous studies, many participants in this study felt prepared and knowledgeable about how to meet CRS needs. Despite this, practical barriers often limited their ability to respond effectively. A key issue was limited access to services outside standard working hours, meaning that even when CRS needs were identified, staff were not always able to act in a timely or appropriate way. This reflects broader evidence that systemic constraints—such as staffing shortages, limited interpreter availability, and reduced access to chaplaincy or community support out of hours—can significantly hinder the delivery of culturally and spiritually responsive care [29,30].

This service evaluation complements the strengths-based perspective, exploring how staff use resources to better understand CRS in PEoLC. It highlights the resourcefulness and commitment of staff in addressing the cultural, religious, and spiritual needs of patients and their families, even in the face of organisational challenges. While earlier studies have tended to focus on gaps in knowledge or preparedness [15,30,31,32], this research demonstrates that many staff members are actively seeking ways to provide responsive and person-centred care.

A key finding is the importance of straightforward access to CRS-related resources for all staff involved in PEoLC. The findings suggest a perceived need for practical tools and guidance to support their work. Participants in this study asked for a CRS-related toolkit that includes: a broad overview of religious and spiritual practices at end of life; online training resources grounded in lived experiences; and easily accessible links to relevant services and community contacts across the LLR area. Such a toolkit would not only support staff in delivering holistic care but also help embed CRS considerations more consistently across PEoLC care. Prototype resources were developed on the basis of this survey and presented to a consultation workshop for feedback. Responses were generally positive, with participants emphasising the value of resources that provide the “basics of religion and cultural needs but not to presume these basics are followed” due to the individualistic nature of religious and cultural practices. However, this prototype toolkit would need formally evaluating to understand its acceptability, implementation and impact.

Staff in this study also expressed concerns that families were not always adequately supported, particularly in relation to accessing information and resources outside standard working hours. This reflects existing literature showing that families receiving, or who have received, PEoLC often experience difficulties accessing out-of-hours services, home-based support, and bereavement care [33]. Participants highlighted limited continuity and accessibility of support beyond their direct care, pointing to wider gaps in community-based provision.

In response, this study argues for the development of a dedicated resource for patients, carers, and bereaved carers across LLR. Such a resource should be co-developed with patients, family members, and bereaved carers and bring together information on relevant community and healthcare services. It could include online materials grounded in lived experience from multiple perspectives, including healthcare professionals, voluntary and community-sector staff, carers, and patients, alongside clear and accessible links to local support services. While this approach has the potential to empower families, improve continuity of care, and support the recognition of CRS needs beyond inpatient settings, formal evaluation would be required.

The findings also align with the existing literature highlighting healthcare professionals’ concerns about unintentionally causing offence or distress when addressing CRS needs, either through omission or through questions, perceived as inappropriate [34]. Participants similarly reported uncertainty and reduced confidence when engaging in CRS-related conversations, particularly within emotionally complex end-of-life contexts. This indicates a need for greater practical support to strengthen staff confidence and communication skills in PEoLC settings.

Islam [34] advocates for an “authentically curious” approach to CRS discussions, emphasising open dialogue and asking rather than assuming. Participants in this study identified training as central to supporting this approach; however, previous research consistently highlights limited access to affordable and accessible CRS-focused training [34]. As discussed earlier, an accessible virtual resource toolkit may help address this gap by collating freely available training materials, practical guidance, lived-experience resources, and local signposting. While promising, the acceptability, implementation, and impact of such a toolkit would require further evaluation.

Finally, feedback from the consultation workshop highlighted support for establishing a dedicated staff forum. This was seen as an opportunity for annual reflection, shared learning, and discussion of cultural and religious challenges in practice, with the aim of fostering ongoing learning and improving the quality of care. Participants suggested that this model could be scaled nationally, creating a forum that brings together professionals from both healthcare and third-sector organisations. A national platform of this kind would strengthen cross-sector collaboration, facilitate the sharing of best practices, and support the ongoing development of culturally and spiritually sensitive care. In doing so, it would contribute to a more consistent and informed approach to meeting CRS needs across diverse care settings.

Importantly, while this evaluation informs the development of a CRS resource toolkit, questions of long-term sustainability warrant consideration. For such a toolkit to remain relevant and responsive, responsibility for its ongoing maintenance, updating of content, and alignment with evolving community needs would need to be clearly established. Without defined ownership or resourcing, there is a risk that tools intended to support practice may become outdated or underused. Future work should therefore explore governance, implementation pathways, and mechanisms for ensuring that co-produced resources remain current, inclusive, and embedded within wider systems of education and support.

### 9.2. Limitations

This study has several important limitations that should be considered when interpreting the findings. First, the sample size was relatively small (*n* = 39), and participants were recruited using purposive and convenience sampling methods. As recruitment relied on distributed sharing through professional and organisational networks, a response rate could not be calculated, and self-selection bias may have influenced participation. Moreover, only 23 participants completed the optional demographic section, and all respondents who provided ethnicity data identified as White. It is therefore not possible to determine whether the full sample reflected the ethnic diversity of Leicester, Leicestershire, and Rutland or whether community, voluntary-sector, non-clinical, and minoritised perspectives were meaningfully represented. This limitation is particularly salient given the study’s location within a highly diverse region.

While this evaluation provides valuable insight into staff perceptions of CRS care, findings must be interpreted in light of potential social desirability bias. Given the professional and normative expectation that culturally and spiritually sensitive practice is integral to good palliative and end-of-life care, participants may have been inclined to frame their responses in ways that reflected positively on their values or practice. As data were entirely self-reported, it is not possible to determine the extent to which expressed confidence or practices translate into routine care delivery. This reinforces the importance of interpreting the findings as perceived confidence and experience, rather than as indicators of competence or quality of care.

A further interpretive tension arises from the study context. Leicester is widely characterised as a “super-diverse” city, and the evaluation was explicitly motivated by this demographic reality. However, all participants who provided demographic information identified as White, and demographic data were missing for a substantial proportion of the sample. This limits the extent to which the findings can be said to reflect the experiences of staff from ethnically diverse backgrounds or those working primarily within culturally specific community settings. This tension has implications not only for the generalisability of the findings but also for how staff accounts of CRS needs are understood, as perspectives may differ according to cultural positioning, lived experience, and professional role. The findings therefore reflect the perspectives of those who engaged with the evaluation and should not be interpreted as representative of the full diversity of the workforce or communities served within LLR.

## 10. Conclusions

As national care quality guidelines and local ICBs continue to advance the ‘dying well’ agenda, there is a growing emphasis on ensuring that PEoLC is personalised and supportive for both patients and families. A key component of this agenda is the recognition that CRS needs must be met as part of holistic care. People accessing PEoLC, and their families through to bereavement, should be well supported not only at the end of life but also beyond. Given that PEoLC is inherently multidisciplinary, requiring collaboration across hospitals, hospices, community organisations, and voluntary and charitable sectors, it is important that CRS perceived confidence and perceived preparedness are strengthened across all staff groups, irrespective of setting.

This service evaluation, conducted across LLR, found that many staff are actively seeking resources to support the delivery of CRS sensitive care; however, these resources were often described as difficult to access and lacking clarity. The findings therefore indicate a perceived need for a CRS focused resource to support staff across PEoLC. Such a resource should be accessible across healthcare, community, and voluntary sector settings and include clear, practical guidance; links to training grounded in lived experience; and signposting to relevant local services. While a prototype toolkit was co-developed in response to these findings, its acceptability, implementation, and impact require further evaluation. Integrating a resource of this kind within existing systems may support more consistent and accessible approaches to CRS-sensitive care, in line with wider policy commitments to equitable and person-centred end-of-life care.

## Figures and Tables

**Table 1:** Inclusion and exclusion criteria.

Inclusion Criteria	Exclusion Criteria
Aged 18 years or over	Aged under 18 years
Currently working or volunteering in any relevant role	Not currently working or volunteering in any relevant role
Role involves supporting people with palliative or end-of-life care needs,	Roles with no involvement in palliative care, end-of-life care, bereavement support, or related community support
Based and Support People in Leicester, Leicestershire and Rutland	Based and Support People outside Leicester, Leicestershire and Rutland area.
Employed or volunteering in any sector (e.g., healthcare, hospice, voluntary, community, charity)	
Any professional discipline, role seniority, contractual status, or length of experience	

**Table 2:** Demographic description of survey participants who provided demographic information.

**Ethnicity**	
White—English/Welsh/Scottish/Northern Irish/British	22
White—Irish	1
**Gender**	
Female	21
Male	2
**Religion/Belief**	
Christian	10
No Religion	8
Buddhist	1
Agnostic	1
Spiritualism	1
Pagan	1
Prefer Not to Answer	1
**Organisation Type**	
Healthcare	14
Registered Charity	6
Other	2
Prefer Not to Answer	1
**Staff Member Job Roles**	
Nurse	11
Medical Staff	7
Clinical Staff	4
Spiritual Worker	1

**Table 3:** Descriptive statistics for Likert scale items assessing cultural, religious, and spiritual (CRS) care.

Item	*n*	*M*	*SD*	Median	IQR
**How often do you consider the cultural needs of people at end of life and their families through to bereavement?**	39	4.48	0.61	4	4–5
**How often do you consider the religious and/or spiritual needs of people at end of life and their families through to bereavement?**	39	4.66	0.53	5	4–5
**Perceived significance of considering cultural needs at end of life and through bereavement**	39	4.82	0.38	5	5–5
**Perceived significance of considering religious and/or spiritual needs at end of life and through bereavement**	39	4.84	0.36	5	5–5
**Confidence asking about religious and/or spiritual needs**	39	3.97	0.74	4	4–4
**Confidence asking about cultural needs**	39	3.82	0.88	4	3–4
**Confidence exploring unfamiliar religious and/or spiritual needs**	39	3.71	0.91	4	3–4
**Confidence exploring unfamiliar cultural needs**	39	3.66	0.92	4	3–4
**Confidence that organisational services can meet religious and/or spiritual needs**	39	3.15	1.11	3	3–4
**Confidence that organisational services can meet cultural needs**	39	3.05	1.16	3	3–4

Note. Items use different response anchors depending on question type: frequency items (1 = Never to 5 = Very often), perceived significance items (1 = Not at all significant to 5 = Very significant), and confidence items (1 = Not confident at all to 5 = Very confident). Items are reported descriptively; no composite scores were calculated.

**Table 4:** Themes of participants’ understanding of cultural and religious/spiritual needs among patients receiving palliative and end-of-life care and their families.

Theme Category	Main Theme	Subthemes	Example Quote
**Understanding of CRS needs**	Holistic and Personalised Care: Importance of Cultural and Religious/Spiritual Needs in PEoLC and beyond		“It’s about recognising the individual as a unique person and understanding what is important to them…”
	Proactive Assessment of Cultural and Religious/Spiritual Needs	Proactively asking patients about their cultural needs	“Cultural needs can be wide ranging—asking people what is important to them…”
		Not Assuming a Person’s Spiritual or Religious Needs	“explicitly ask them whether there is something I need to be made aware of in relation to this.”
	Understanding Cultural Identity of Patients and Families	Culture Goes Beyond ‘Religion’ and ‘Ethnicity’	“My understanding of cultural needs is not just about religion…”
		Importance of Dietary Needs seen as ‘vital’ to culture	“Representing their dietary needs, language and acknowledging their practices”
	Religious/Spiritual Practices of Patients and their Families	Rites, Rituals and Prayers	“Some religions have ceremonies… Muslims would opt for positioning towards Mecca…”
**Barriers, Concerns, and Practical Challenges**	Role of Family and Community in Supporting Patients with a PEoLC Need and their Families through to Bereavement	Role of Friends and Family in Cultural Practices and Decisions	“Families may opt for an open casket and welcome visitors…”
		Role of Friends and Family in Spiritual/Religious Care and Bereavement	“The family will relive this time many times and knowing that everything possible is done will help them with their bereavement”
	Impact of Unmet Cultural and Spiritual/Religious Needs		“Unmet religious or spiritual needs can lead to a great deal of distress at an already emotional time for patients and families.”
	Fear of Causing Offence		“My main concern is about unintentionally causing offence or distress by asking questions in an insensitive way, especially during such a vulnerable time.”
**Resources, Training Needs, and Support**	Use of existing resources		“chaplaincy, education, palliative care, bereavement support.”
	Need for accessible training and guidance		“broad overview of traditions required for specific religious needs”

## Data Availability

A fully anonymized raw dataset looking at Likert responses and some open-ended answers (services available and other factors) will be available as Supplementary Materials. No direct identifiers were requested; however, open-text responses were reviewed for potential indirect identifiers before analysis and data sharing.

## Supplementary Materials

The following supporting information can be downloaded at: https://www.mdpi.com/article/10.3390/healthcare14111555/s1, Section S1: Understanding Cultural, Religious and Spiritual Needs in End of Life Care through to bereavement. Section S2: Demographics.

## References

[1] Carroll B. (2001). A phenomenological exploration of the nature of spirituality and spiritual care. Mortality.

[2] Martins H., Romeiro J., Casaleiro T., Vieira M., Caldeira S. (2024). Insights on spirituality and bereavement: A systematic review of qualitative studies. J. Clin. Nurs..

[3] Balboni T.A., Balboni M., Enzinger A.C., Gallivan K., Paulk M.E., Wright A., Steinhauser K., VanderWeele T.J., Prigerson H.G. (2013). Provision of spiritual support to patients with advanced cancer by religious communities and associations with medical care at the end of life. JAMA Intern. Med..

[4] Jackson D., Doyle C., Capon H., Pringle E. (2016). Spirituality, spiritual need, and spiritual care in aged care. J. Relig. Spiritual. Aging.

[5] National Institute for Health and Care Excellence (2019). End of Life Care for Adults: Service Delivery (NG142).

[6] Gunawardena N., Britton H., Roy J., Harding S., Eckoldt S., Lovell N. (2024). Improving the assessment of cultural, religious, and spiritual needs at end of life. BMJ Open Qual..

[7] Lazenby M. (2018). Understanding and addressing the religious and spiritual needs of advanced cancer patients. Seminars in Oncology Nursing.

[8] Samuels A., Lemos Dekker N. (2023). Palliative care practices and policies in diverse socio-cultural contexts: Aims and framework of the ERC globalizing palliative care comparative ethnographic study. Palliat. Care Soc. Pract..

[9] Strang S., Strang P., Ternestedt B.M. (2002). Spiritual needs as defined by Swedish nursing staff. J. Clin. Nurs..

[10] Department of Health and Social Care (2025). Fit for the Future: 10 Year Health Plan for England.

[11] Marie Curie Palliative and End of Life Care Policy Briefing. https://www.mariecurie.org.uk/document/terminally-ill-adults-parliamentary-briefing.

[12] Hospice UK Specialist Community Hospice Visits Fall by 150,000 in a Year. https://www.hospiceuk.org/latest-from-hospice-uk/specialist-community-hospice-visits-fall-150000-year.

[13] Dillard V., Moss J., Padgett N., Tan X., Kennedy A.B. (2021). Attitudes, beliefs, and behaviors of religiosity, spirituality, and cultural competence in the medical profession: A cross-sectional survey study. PLoS ONE.

[14] Russell J., Quaack K.R., Nunez J. (2023). Chaplain reported plans for end of life conversations. J. Health Care Chaplain..

[15] O’Brien M.R., Kinloch K., Groves K.E., Jack B.A. (2019). Meeting patients’ spiritual needs during end of life care. J. Clin. Nurs..

[16] Cáceres Titos M.J., Porras Santana J.M., Cabillas Romero M.R., García Navarro E.B. (2025). Managing cultural diversity in end of life care: A qualitative study. BMC Palliat. Care.

[17] Kincheloe D.D., Stallings Welden L.M., White A. (2018). A spiritual care toolkit: An evidence-based solution to meet spiritual needs. J. Clin. Nurs..

[18] Sunga P. (2021). Implementing an Educational Toolkit on Spirituality for Nursing Staff in Outpatient Oncology Clinics. Ph.D. Thesis.

[19] Liu Y.-J. (2014). A proposal for a spiritual care assessment toolkit for religious volunteers and volunteer service users. J. Relig. Health.

[20] Office for National Statistics How Life Has Changed in Leicester: Census 2021. https://www.ons.gov.uk.

[21] Smith H., Budworth L., Grindey C., Hague I., Hamer N., Kislov R., Van Der Graaf P., Langley J. (2022). Co-production practice and future research priorities. Health Res. Policy Syst..

[22] Filipe A., Renedo A., Marston C. (2017). The co-production of what? Knowledge, values, and social relations in health care. PLoS Biol..

[23] Vargas C., Whelan J., Brimblecombe J., Allender S. (2022). Co-creation, co-design, and co-production for public health. Public Health Res. Pract..

[24] Masterson D., Areskoug Josefsson K., Robert G., Nylander E., Kjellström S. (2022). Mapping definitions of co-production and co-design. Health Expect..

[25] IBM Corp (2020). IBM SPSS Statistics for Windows.

[26] Braun V., Clarke V. (2021). Thematic Analysis: A Practical Guide.

[27] Lumivero (2025). NVivo.

[28] Glyn Blanco M.B., Lucchetti G., Badanta B. (2023). How do cultural factors influence the provision of end of life care?. Appl. Nurs. Res..

[29] VanderWeele T.J. (2017). Religion and health: A synthesis. Spirituality and Religion Within the Culture of Medicine.

[30] Sheikh A., Gatrad A.R. (2021). Death and bereavement. Caring for Muslim Patients.

[31] Crawley L.M., Marshall P.A., Lo B., Koenig B.A. (2002). Strategies for culturally effective end of life care. Ann. Intern. Med..

[32] Fang M.L., Sixsmith J., Sinclair S., Horst G. (2016). A knowledge synthesis of culturally and spiritually sensitive end of life care. BMC Geriatr..

[33] Hodge G., Kallis G., Oh T.M., Wheat H., Pearce S. (2025). Exploring perceived barriers to palliative and end of life care provision. Front. Sociol..

[34] Islam Z., Pollock K., Patterson A., Hanjari M., Faull C. (2024). An exploration of cardiopulmonary resuscitation discussions with ethnically diverse patients and families. Br. J. Card. Nurs..

