# Supplementary material for: Assessing Cultural, Religious, and Spiritual Confidence and Perceived Preparedness in Community Palliative and End-of-Life Care: A Service Evaluation
Source: Healthcare (Basel). 2026 Jun 2;14(11):1555. doi: 10.3390/healthcare14111555 (PMC13257279; doi:10.3390/healthcare14111555)
Supplement: Supplementary file 1 [file healthcare-14-01555-s001.zip › healthcare-4281958-supplementary.pdf]

Cultural, Spiritual and Religious Needs of People who are Dying and their Families

Time to Complete: 15 Minutes

Blurb: Thank you for taking the time to participate in our survey. We are dedicated to better understanding and enhancing the spiritual, religious, and cultural care provided to patients at the end of life, as well as the support available to their families during bereavement.

This questionnaire is split into two sections. Section 1 focuses on the confidence and competence of your organisation/service in providing culturally, religiously, and spiritually sensitive care to people receiving palliative and/or end of life care, as well as their families.Section 2 aims to collect some demographic information, but is completely optional*.

*We are collecting demographic data because we recognize that inequalities and inequities exist in palliative, end of life, and bereavement care. Understanding who works in these settings helps us identify gaps and move toward more equitable and inclusive care practices.Thank you for your participation and contribution to this important effort.

Section S1: Understanding Cultural, Religious and Spiritual Needs in End of Life Care through to bereavement

In this section, we aim to gather insights on your experiences and perspectives regarding the cultural, religious, spiritual needs of dying patients and their families at end of life and post-death (bereavement). Your responses will help us understand how these needs are recognised and addressed within your role and the wider organisation. Please feel free to put N/A as an answer if you don't want to answer a question.

1. What is your understanding of the cultural needs of people receiving palliative and end-of-life care, as well as the needs of their families through to bereavement?
2. What is your understanding of the spiritual and religious needs of people receiving palliative and end-of-life care, as well as the needs of their families through to bereavement?
3. How often do you consider the cultural needs of people at end of life and their families through to bereavement?
    a. Never
    b. Rarely
    c. Sometimes
    d. Often
    e. Very Often
4. How often do you consider spiritual/religious needs of people at end of life and their families through to bereavement? Required to answer. Single choice.
    a. Never
    b. Rarely
    c. Sometimes
    d. Often
    e. Very Often
5. In your opinion, how significant is it to consider cultural needs for a person receiving palliative and end of life care and their family, through to bereavement? (e.g. end of life /bereavement support)
    a. Not Significant
    b. Slightly Significant
    c. Neutral

    d. Very Significant
    e. Extremely Significant
6. In your opinion, how significant is it to consider the **spiritual and/or religious needs** for a person receiving palliative and end of life care and their family (e.g. end of life /bereavement support)
    a. Not Significant
    b. Slightly Significant
    c. Neutral
    d. Very Significant
    e. Extremely Significant
7. The following set of statements aims to understand how confident you feel in addressing the cultural, spiritual and religious needs of patients receiving palliative and end-of-life care, as well as the needs of their families through to bereavement. Please rate each statement below based on your level of confidence, where 1 indicates not very confident and 5 indicates incredibly confident. Required to answer. Likert.
    a. How confident do you feel in asking about religious and/or spiritual needs of a person receiving palliative or end of life care and their family through to bereavement?
    b. How confident do you feel in asking about cultural needs of a person receiving palliative or end of life care and their family through to bereavement?
    c. If a person, receiving palliative and/or end of life care or their families express religious and/or spiritual needs that you are not familiar with, how confident do you feel in exploring how these needs can be met in the hospital, hospice or community?
    d. If a person, receiving palliative and/or end of life care or their families express cultural needs that you are not familiar with, how confident do you feel in exploring how these needs can be met in the hospital, hospice or community?
    e. How confident do you feel in the services and facilities of the wider hospital/hospice or community to meet the religious/cultural/spiritual needs of patients at the end of their lives and post-death (bereavement support services)?
8. Please describe how supported you feel within the organisation/service you work for to meet the religious/cultural/spiritual needs of patients at the end of life and their families through to bereavement?
9. What worries or concerns do you have about asking people who are dying or their families about their cultural/religious /spiritual needs?
10. What practical challenges do you face, if any, when addressing cultural/ religious /spiritual needs in dying patients and their families?
11. Are you aware of any services or teams at your organisation or other organisations that provide support in addressing religious/cultural/spiritual needs of patients at the end of life and their families? Required to answer. Single choice.
    a. Yes
    b. No
    c. If you answered yes, please provide the details of which services or teams which could provide support to you?
12. Have you received any training on addressing religious/cultural/spiritual needs of dying patients and their families through to bereavement?
    a. Yes
    b. No

   c. If you answered yes, please could you provide any training you have had on addressing religious/cultural/spiritual needs of dying patients and their families through to bereavement?
13. Would you consider attending training sessions on addressing religious/cultural/spiritual needs of dying patients and their families, through to bereavement, if they were provided? Required to answer. Single choice.
    a. Yes
    b. No
    c. Maybe
    d. If you answered yes or maybe, please could you provide some more details about what you feel would be useful to have in the training sessions about addressing cultural/spiritual/religious needs of patients at the end of life and their families?
14. If you answered no, please could you explain why you felt this way?
15. What training/resources do you currently access to help you to support patients and their families in meeting their religious/cultural/spiritual needs?
16. What resources and/or training do you think is currently needed to support you in meeting the religious/cultural/spiritual needs of patients at the end of life and their families through to bereavement? Is there anything you'd like to add?

Section S2: Demographics

This section is entirely optional. If you do not wish to complete this section please tick the box below and it will move you to the end of the survey. This section looks at collecting demographic data to better understand the diverse backgrounds and experiences of our participants. Please feel free to provide a much or as little information as you feel comfortable with sharing.

1. How old are you (in years, please use numbers only e.g. 24)
2. What is your gender?
    a. Female
    b. Male
    c. Prefer Not to Say
    d. Other
3. Is your gender identity the same as the sex you were registered at birth?
    a. Yes
    b. No
    c. Prefer Not To Say
4. What is your ethnic group?
5. What is your country of Birth?
    a. UK
    b. Prefer Not To Say
    c. Other
6. What year did you first come to live in the UK? Please provide the year
7. What is your first language (e.g. the language you learned first or the only language you speak with your family?)
    a. English
    b. British Sign Language
    c. Other
8. Do you speak any other languages, if yes please specify?
9. What is your religion/belief?

a. Buddhist
b. Christian
c. Hindu
d. Humanist
e. Jewish
f. Muslim
g. No Religion
h. Sikh
i. Prefer Not To Answer
j. Other

10. Below is a list of organisation types, please could you choose which option best applies to you?
    a. Registered Charity
    b. Educational Institution (University, Primary/Secondary School)
    c. Healthcare Organisation (e.g. Primary Care (Acute hospitals, Mental Health Hospitals, Private Clinics))
    d. Community Organisation (e.g. Youth Club, Local Community Centre, Mental Health Support Groups)
    e. Private Sector (e.g. Retail, Financial Services)
    f. Public Sector (e.g. Local Council, National Government Department)
    g. Prefer Not to Answer
11. Please could you specify your job role within your organisation (Clinical and Non-Clinical)?
12. Please specify how many years of experience you have in the role you just identified in the question above?
    a. Less than three years
    b. More than three years

END OF SURVEY
